# Sociodemographic and disease-related determinants of return to work among women with breast cancer: a German longitudinal cohort study

**DOI:** 10.1186/s12913-018-3768-4

**Published:** 2018-12-29

**Authors:** Christian Heuser, Sarah Halbach, Christoph Kowalski, Anna Enders, Holger Pfaff, Nicole Ernstmann

**Affiliations:** 1 Center for Health Communication and Health Services Research (CHSR), Department for Psychosomatic Medicine and Psychotherapy, University Hospital Bonn, Sigmund-Freud-Str. 25, 53127 Bonn, Germany; 2 Center for Integrated Oncology (CIO Bonn), University Hospital Bonn, Sigmund-Freud-Str. 25, 53127 Bonn, Germany; 3 German Cancer Society e.V. (DKG), Department for Certification, Kuno-Fischer-Straße 8, 14057 Berlin, Germany; 4 The Federal Centre for Health Education (BZgA), Department for Research and Quality Management, Maarweg 149-161, 50825 Cologne, Germany; 5 Institute of Medical Sociology,Health Services Research, and Rehabilitation Science (IMVR), Faculty of Human Sciences and Faculty of Medicine, University of Cologne, Eupener Str. 129, 50933 Cologne, Germany

**Keywords:** Return to work, Vocational rehabilitation, Occupational rehabilitation, Breast cancer, Health services research

## Abstract

**Background:**

Return to work (RTW) is a key parameter of outcome quality that ensures social participation. Therefore, this study analyses the sociodemographic and disease-related determinants of RTW among newly diagnosed breast cancer patients.

**Methods:**

In a prospective, multicentre cohort study, breast cancer patients were surveyed three times: directly after surgery, after 10 weeks, and after 40 weeks. Logistic regression analysis was applied to estimate the association of RTW at 40 weeks following discharge with sociodemographic and disease-related characteristics (*n* = 577).

**Results:**

The sociodemographic variables “entrance certificate at a university of applied science” compared to “university entrance certificate” (OR = 3.1, 95%-CI = 1.2–8.1), age group “55–59 years” compared to “18–44 years” (OR = 3.2, 95%-CI = 1.2–8.4) and “having children” (OR = 2.8, 95%-CI = 1.2–6.2) as well as the disease-related variables “rehabilitation” (OR = 0.5, 95%-CI = 0.3–0.9), self-rated health “good” and “excellent” compared to “bad” (OR = 2.7, 95%-CI = 1.4–5.5; OR = 11.6, 95%-CI = 4.2–31.8) and the UICC-classification “stage II” and “stage III/IV” in comparison to “stage 0/I” (OR = 0.5, 95%-CI = 0.3–0.8; OR = 0.2, 95%-CI = 0.1–0.5) significantly affect RTW among breast cancer patients (Nagelkerke’s Pseudo-R^2^ = 0.275).

**Conclusions:**

The findings show that significant differences in RTW exist between patient groups and suggest that RTW issues must be addressed more effectively before, during and after treatment. For future research on RTW in Germany, longitudinal studies with a follow-up of several years are necessary. Information and support deficits should be tackled by social services or breast care nurses.

**Trial registration:**

Database Health Services Research, VfD_PIAT_12_001630, registered 01.03.2012

**Electronic supplementary material:**

The online version of this article (10.1186/s12913-018-3768-4) contains supplementary material, which is available to authorized users.

## Background

Return to work (RTW) following an oncological disease represents a major challenge for patients. The scale of physical and emotional coping and impaired physical functioning amongst patients often results in reduced working hours, amended tasks, exhaustion or unemployment [1–3]. A large number of international studies have investigated structural health inequalities, including in vocational and social reintegration, such as in the case of breast cancer care [4–8]. However, in Germany only a few studies address RTW [9, 10], thus creating a research gap regarding breast cancer. With an incidence of 71,600 women in 2013, breast cancer is the most common oncological disease in Germany [11]. Moreover, the incidence rate shows an increase of 1.3%, which is slightly higher than the European Union (EU) average [11]. The 5- and 10-year survival rate stands respectively at 88 and 82%, and the trend of deaths is showing a decline of 1.2%, resulting in growing prospects of surviving breast cancer in comparison to other oncological diseases. Improved therapy options and a high survival rate have rendered RTW a highly pertinent issue for women with breast cancer.

Bringing the mentioned aspects and studies together we define RTW as a complex process containing coping, physical and mental health, sense of normality, quality of life, financial security and social participation. Furthermore, international approaches have identified five influential categories concerning RTW in the case of breast cancer which we follow in this study: diagnosis and medical factors [12]; treatment and factors pertaining to functional status [13]; work-related factors [14, 15]; psychosocial factors [16]; and sociodemographic factors [5, 6, 8] such as formal education, income, age, health insurance status and lifestyle [17, 18]. Based on the international state of research shown below, several research hypotheses can be developed for this study.

### State of research and hypotheses

i) Low levels of formal education are associated with a lower chance of RTW [5, 7, 19–21]. Kolodziejczyk et al. [22] found an educational gradient for the public and private sector in terms of labour market participation among women with breast cancer. Having a secure and well-paid job prior to the disease has an important impact on RTW because an insecure employment situation may be exacerbated following the disease [23]. In particular, post-disease unemployment is associated with a lower level of formal education as well as either starting a different job than that prior to the disease or resorting to early retirement [2]. Education-based inequalities in RTW can also be significant in relation to physician-patient interaction, as the likelihood of a follow-up discussion for less educated breast cancer patients can be lower than is true of highly educated women [24]. This result is consistent with other research from Germany, which finds that low levels of education are associated with a greater number of unmet information needs during hospitalisation [25] and rehabilitation [26]. As the first hypothesis it can be assumed that greater education promotes RTW.

ii) Age is also associated with RTW [19, 27]. Concerning the direction of impact, it has been shown that older age leads to less work [28], unemployment [1, 5] or early retirement [7]. As the second hypothesis it can thus be assumed that younger age is positively correlated with RTW.

iii) A large number of studies have concluded that single women return to work significantly more often than married women, often as a result of financial necessity, whereas married women can often rely on a degree of financial security through their spouse [29, 30]. As a third hypothesis, it can hence be assumed that single women return to work more often.

iv) Closely connected with the third hypothesis are results from numerous studies which found out that (single) women with children return to work more often than women without children, often due to financial necessity [30, 31]. As a fourth hypothesis, having children might be associated with a higher likelihood for return to work.

v) The state of research regarding ethnicity and RTW for Germany is rudimentary. In general, international evidence suggests that belonging to an ethnic minority represents a barrier to RTW [30, 32]. Therefore, as a fifth hypothesis, having an ethnic minority background seems to be a barrier for RTW.

vi) According to previous research, health insurance status does not unequivocally influence the care of breast cancer patients. Germany is frequently confronted with the question of whether differences exist between statutory and private care services and a related lack of knowledge concerning RTW. Therefore, the direction of the effect of the sixth hypothesis is not to be formulated.

vii) Finally, it is helpful to consider further studies for Germany, especially regarding the effect of general cancer rehabilitation on RTW [14]. Until now, RTW among breast cancer patients in Germany has only been investigated by Noeres et al. [9], who found that rehabilitation had a positive effect on RTW. Studies combining rehabilitation, sociodemographic and disease-related factors are absent. The last hypothesis concerning the association of rehabilitation and RTW is also formulated without a direction of the effect.

It has been argued that international studies regularly suggest an association between RTW and breast cancer patients’ sociodemographic characteristics, or between RTW and disease-related factors. In this study the combination of sociodemographic and disease-related factors in a multivariate statistical model may contribute to the international body of research on RTW. Based on the current state of research, two main research questions can be formulated: 1) What kind of work changes, e.g. work time, tasks, income or stress, can be described after breast cancer care and who has supported patients concerning RTW (descriptive results)? 2) How are the aforementioned six sociodemographic determinants associated with RTW when adjusting for disease-related variables (multivariate results)?

## Methods

### Study design and sample

The prospective, longitudinal, multicentre cohort study “PIAT”^1^ was conducted in German breast cancer centres. Data were collected from 2013 to 2014 using written questionnaires with standardised mainly self-report measures. A random sample of 98 out of 247 German breast cancer centres that are certified as meeting the criteria of the German Cancer Society and the German Society for Senology were invited to participate. Finally, 56 out of the 98 breast cancer centres took part in the study whereas the rest declined to participate. Breast cancer centers characteristics were: nationwide geographical location, 90% teaching hospitals, 46.1% public ownership, 42.4% free profit ownership, 11.5% private ownership, 0.2–6.2% range of percentage from patients in centers. Patients’ inclusion criteria comprised inpatient surgery for newly diagnosed breast cancer (C50.xx, D05.xx) between February 1 and August 31 2013, and at least one malignancy and at least one postoperative histological evaluation. After providing written informed consent patients filled out an initial questionnaire during the hospital stay directly following breast cancer surgery (T1, response rate = 87.7%, *N* = 1359). The same patients were surveyed again with two postal surveys conducted 10 weeks (T2, during follow-up treatment, *N* = 1151) and 40 weeks after their breast cancer surgery (T3, after post-treatment, *N* = 1060). The survey was designed according to Dillman’s Total Design Method, with three reminders being made [33]. In the present work, the data of *N* = 577 women were considered: these women were employed (full- or part-time or vocational rehabilitation programme) or had the self-reported capability of employment but were currently certified sick by breast cancer centre or rehabilitation centre. No imputations were performed for missing data. Cases with missing data for the dependent variable were excluded.

### Instruments and variables

Data regarding sociodemographic patient characteristics were assessed in the patient survey at T1. Data regarding clinical patient characteristics were provided by clinical personnel (mainly breast care nurses, oncologists) at T1 (see Table 1) based on chart review. Clinical patient characteristics served as control variables in the regression analysis. Data on RTW were assessed at T3, as well as participation in a rehabilitation programme (yes/no). Changes in work conditions were measured with self-reported items which were “no” or “yes” coded (for the items see Additional file 1: Table S4). Support concerning RTW was measured with self-reported items which were “no” or “yes” coded (see Table 2). Regarding the dependent variable RTW, patients were asked “Have you continued your previous professional activity unchanged?” (yes/no). All measures and instruments used in the questionnaires were pre-tested in a pilot study using interviews and focus groups with breast cancer patients and experts as described elsewhere [25, 34]. Table 1 shows the descriptive results of the *N* = 577 patients included in the model. No multicollinearity was found between the independent variables.

**Table 1:** Descriptive results of the used variables (*N* = 577)

Variables	Response trait	*n* (%)
Dependent variable: return to work	No	203 (35.2)
	Yes	374 (64.8)
Independent variables: Sociodemographic variables		
Highest education level achieved	No/Lower secondary school education	333 (57.7)
	Intermediate secondary school education/Entrance certificate for a university of applied sciences	108 (18.7)
	University entrance certificate	92 (16.0)
	Missing	44 (7.6)
Age	18–44	86 (14.9)
	45–49	128 (22.1)
	50–54	155 (26.9)
	55–59	106 (18.4)
	≥60	71 (12.3)
	Missing	31 (5.3)
Family status	Married	372 (64.5)
	Single	65 (11.3)
	Divorced/Widowed	108 (18.7)
	Missing	32 (5.5)
Children	No	112 (19.4)
	Yes	432 (74.9)
	Missing	33 (5.7)
Native language	German	519 (89.9)
	Other	28 (4.9)
	Missing	30 (5.2)
Health insurance status	Statutory	418 (72.5)
	Private	56 (9.7)
	Statutory with additional private insurance	71 (12.3)
	Missing	32 (5.5)
Disease-related variables		
Rehabilitation	No	147 (25.5)
	Yes	256 (44.4)
	Missing	174 (30.1)
Individual health status	Bad/Less good	104 (18.0)
	Good	332 (57.5)
	Very good/Excellent	136 (23.6)
	Missing	5 (0.9)
Comorbidities	0	49 (8.5)
	1	425 (73.6)
	2	48 (8.3)
	3	20 (3.5)
	4	4 (0.7)
	5	1 (0.2)
	Missing	30 (5.2)
ASA classification	1	291 (50.4)
	2	210 (36.4)
	3	17 (3.0)
	4–6 was not reported	0 (0)
	Missing	59 (10.2)
UICC stage	Stage 0/I	256 (44.4)
	Stage II	178 (30.8)
	Stage III/IV	53 (9.2)
	Missing	90 (15.6)

### Analysis

First, the frequencies of RTW and sociodemographic variables were analysed using descriptive statistics. Second, to answer the first research question, work changes after breast cancer care were analysed descriptively comparing subgroups with different sociodemographic characteristics. Third, intercorrelations among the independent variables were checked for multicollinearity by calculating Pearson or Spearman correlation coefficients. Lastly, logistic regression modelling was applied, which facilitates estimation of the aforementioned patient characteristics as predictors for RTW, with the help of the maximum likelihood method. All statistical analyses were conducted in IBM SPSS Statistics version 24.

## Results

### Descriptive results

In order to answer the first research question, changes in work conditions are presented. 203 (35.2%) patients did not return to work within 40 weeks of surgery, whereas 374 (64.8%) patients did return to work. The latter patients with successful RTW (*n* = 374) reported changed working conditions that differed between subgroups (for the items see Additional file 1: Table S4). A common pattern can be observed in the case of RTW (*n* = 374): after breast cancer surgery, a reduction in women’s working time was seen (13.2%), resulting in reduced income (7.5%) and less stress (10.1%). An amended range of tasks (6.1%) or employer (2.8%) might also arise. In particular, subgroup differences were observed between women with higher and lower levels of education, and across different age groups: highly educated women worked significantly reduced hours, as did young women aged 18–44 years, who also felt less stressed than women aged 50–65 years. Indeed, in most cases women aged 50–65 did not reduce their working time.

Women with successful RTW (n = 374) but without altered working conditions reported information in support of RTW. A common pattern of RTW-supporting professionals and institutions can be observed, as many women did not receive any support (28.1%), 34.7% obtained support from their employer, whereas health and pension insurance were far less frequently mentioned (17.0%; 5.9%). Support from rehabilitation institutions was reported by 18.0% of the 374 patients. Differences in subgroups occurred especially between those with contrasting levels of education and from different age groups: support from rehabilitation institutions was more often mentioned by highly educated women, whereas support from employers was far more commonly cited by women with intermediate secondary school education/entrance certificate for a university of applied sciences (47.3%). Concerning age-related differences, it can be reported that the age groups 45–49, 50–54 and 55–59 years received more support from employers, rehabilitation institutions or health and pension insurance than did those in the age group 18–44 years. Support from employers reached the highest values (41.5% in 55–59; 38.7% in 50–54; 40.6% in 45–49; 22.1% in 18–44), although rehabilitation institutions were also supportive (19.8% in 55–59; 19.4% in 50–54; 21.1% in 45–49; 16.3% in 18–44). Table 2 shows the descriptive results of support in RTW of women with successful RTW (*n* = 374) but without altered working conditions.

**Table 2:** Descriptive results of support in RTW of women with successful RTW (*n* = 374)

Who has supported you concerning RTW? (in %, multiple replies possible)		No support	Health insurance	Pension insurance	Employer	Rehabilitation institution
		28.1	17.0	5.9	34.7	18.0
Highest education level achieved	No/Lower secondary school education	28.8	18.0	5.1	33.6	17.7
	Intermediate secondary school education/Entrance certificate for a university of applied sciences	23.1	18.5	6.5	47.9	17.6
	University entrance certificate	33.7	12.0	7.6	33.7	21.7
Age	18–44	31.4	14.0	2.3	22.1	16.3
	45–49	25.8	21.9	5.5	40.6	21.1
	50–54	21.3	16.1	8.4	38.7	19.4
	55–59	30.2	22.6	8.5	41.5	19.8
	≥60	39.4	5.6	2.8	21.1	11.3

### Multivariate results

Concerning sociodemographic characteristics, the model showed that patients with intermediate secondary school education or an entrance certificate for a university of applied sciences returned to work more often (OR = 3.10; 95%-CI = 1.19–8.07) than patients with a university entrance certificate. Patients aged 55–59 were also more likely to return to work compared with patients aged 18–44 (OR = 3.21; 95%-CI = 1.22–8.42). Women with children returned to work more often (OR = 2.77; 95%-CI = 1.24–6.19) than women without children. Concerning disease-related variables, participation in a rehabilitation programme was associated with a reduced chance of returning to work amongst women with breast cancer (OR = 0.49; 95%-CI = 0.27–0.92). A good (OR = 2.73; 95%-CI = 1.36–5.47) and very good/excellent (OR = 11.57; 95%-CI = 4.2–31.84) self-reported individual health status promoted a return to work in comparison with poor or inferior health. Finally, the UICC stage II (OR = 0.45; 95%-CI = 0.25–0.82) and stage III/IV (OR = 0.19; 95%-CI = 0.08–0.46) represent barriers to returning to work compared with stage O/I. Pseudo-R^2^ of Nagelkerke and McFadden was used to verify the logistic regressions model. Nagelkerke’s Pseudo-R^2^ assumes a value of .275 and McFadden’s Pseudo-R^2^ of .171. Table 3 shows the results for the logistic regression model for RTW.

**Table 3:** Logistic regression model with return to work as the dependent variable^1^

Variables	Response trait	OR^2^	95%-CI^3^
Sociodemographic variables			
Highest education level achieved	No/Lower secondary school education	1.91	0.85–4.29
	Intermediate secondary school education/Entrance certificate for a university of applied sciences	**3.10**	**1.19–8.07**
	University entrance certificate	1.00	
Age	18–44	1.00	
	45–49	1.46	0.63–3.38
	50–54	1.57	0.64–3.83
	55–59	**3.21**	**1.22–8.42**
	≥60	1.67	0.63–4.43
Family status	Married	1.00	
	Single	1.04	0.36–3.01
	Divorced/Widowed	0.94	0.47–1.88
Children	No	1.00	
	Yes	**2.77**	**1.24–6.19**
Native language	German	1.00	
	Other	1.28	0.38–4.28
Health insurance status	Statutory	0.63	0.23–1.78
	Private	1.00	
	Statutory with additional private insurance	0.42	0.13–1.37
Disease-related variables			
Rehabilitation	No	1.00	
	Yes	**0.49**	**0.27–0.92**
Individual health status	Bad/Less good	1.00	
	Good	**2.73**	**1.36–5.47**
	Very good/Excellent	**11.57**	**4.2–31.84**
Comorbidities	Metric (0–5)	0.90	0.59–1.36
ASA classification	Metric (1–4)	1.12	0.67–1.90
UICC stage	Stage 0/I	1.00	
	Stage II	**0.45**	**0.25–0.82**
	Stage III/IV	**0.19**	**0.08–0.46**

Note: ^1^significant results in bold (*p* < 0.05); ^2^standardized odds ratio (OR); ^3^95% confidence intervals (95%-CI)

## Discussion

This study aimed to examine 1) work changes following breast cancer care and 2) how sociodemographic characteristics are associated with RTW when adjusting for disease-related variables. The logistic regression model showed that RTW among newly diagnosed breast cancer patients differed depending on the patients’ sociodemographic and disease-related characteristics, albeit not necessarily in the direction the state of research expected.

Most surprisingly, women with a university entrance certificate demonstrated the lowest likelihood of RTW compared with all other levels of education. Several reasons might account for this finding. First, in population-based longitudinal studies it has been shown that the effects of education fluctuate over a period of 30 years, or disappear for a limited period of time [35, 36]. This means that in a given period of time, a higher level of education leads to a higher likelihood of RTW, even if in other periods this education instead becomes a barrier to RTW. This potential explanation seems to be plausible given that 40 weeks following surgery represents only a short follow-up time. Different studies internationally have highlighted that a 2-, 5- or even 10-year follow-up following surgery is necessary in order to identify significant results regarding RTW [9, 37–39]. Second, some studies show that certain tasks in jobs requiring only a lower level of education are physically more exhausting. However, this does not necessarily lead to the impossibility of work, as the descriptive results in this study highlight the opposite effect: less educated women work the same amount, whereas highly educated women reduce their work time or do not work at all. The descriptive results also show that support from employers constitutes the most important supportive factor, yet it is mentioned significantly more often by women with an intermediate secondary school education. This group also shows significant results in the multivariate logistic model, which reveals the importance of work-related factors. Third, individual or household income was not taken into account, which means that the statistical model was not corrected for the relationship between education and income. Many studies have shown that women with a low income are more likely to be forced to work again following breast cancer surgery [38]. Lastly, some qualitative studies have investigated different coping strategies amongst highly educated women [40]. The possible altered sense-giving components of women’s lives could thus shift from work to family and health. These reasons might cause the results to be significant but oriented in the opposite direction from that predicted in hypothesis 1.

In hypothesis 2 we suggested that young patients favour RTW. This has not been replicated in the findings. As a post-hoc comparison has shown, 18% of the youngest age group (18–44 years) had a stage III or IV, which is by far the highest value among all age groups. As other studies have demonstrated [5, 41], this significantly higher proportion of serious diagnoses leads to a reduced likelihood of reintegration owing to often-necessary chemotherapy. Furthermore, as young women may develop a higher UICC stage than older women, RTW is less feasible [11]. Descriptive results show that patients aged 18–44 years worked reduced hours or not at all, whereas women aged 50–65 years did not reduce their work time. In addition, people in the age groups 45–49, 50–54 and 55–59 years received more support from their employers, rehabilitation institutions and health and pension insurance than did those in the age group 18–44 years, supporting the multivariate finding. In this study, “having children” also appeared to be relevant. 75% of women aged 18–44 years had at least one child and 74% lived together with their partner; factors that can provide greater economic security. In addition to the aforementioned disease-related decision against RTW, the sociodemographic variables age, having children and family status intensified the effect for the 18–44-year-old patients as 51.2% did not return to work. In contrast, the disease-related factors of patients aged 50–65 years appeared to be less important as the UICC-stage was significantly lower and sociodemographic factors supported RTW as their children were no longer living in the household. Lastly, given that 21% of the patients aged 50–65 years were divorced or widowed, it can be assumed that older patients might feel greater economic necessity to RTW.

In the third hypothesis we assumed that single breast cancer patients were significantly more likely to return to work than their married counterparts. This effect could be seen descriptively, especially with regard to the subgroups of highly educated and young individuals, although it is not significant in the model. Therefore, this hypothesis must be rejected.

Our assumption in the fourth hypothesis that children increase the likelihood of patients returning to work can be confirmed. In addition, the model presented here contributes to a form of differentiation as 75% of the patients aged 18–44 years had children but were less likely to return to work (see non-significant hypothesis 2). On the one hand, the disease-related determinant “UICC stage” was significantly higher. On the other hand, these women revealed the sociodemographic characteristics “married”, “living with their partner” and “having children”, which collectively render return more unlikely. This differentiation of the effect might represent a strength of the model presented.

In hypothesis 5 we assumed a link between ethnic minority status and RTW in accordance with international studies. Through the operationalisation of ethnicity in terms of mother tongue, no significant connection was found. Hypothesis 5 must therefore be rejected.

In hypothesis 6 we suggested an effect of health insurance status amongst breast cancer patients, but no direction was given. Descriptive differences were found as patients with statutory insurance and additional private insurance had a lower likelihood of RTW than did patients with private insurance. However, in the logistic model the effect was not significant. Hypothesis 6 can be rejected.

Our assumption in hypothesis 7 was largely based on results from a previous study in Germany that rehabilitation would favour RTW. However, in the logistic regression model a significant but negative effect was found. Indeed, rehabilitation was associated with a lower likelihood of RTW. In contrast, the descriptive results showed that working patients following surgery (*n* = 374) said that rehabilitation institutions were supportive as regards RTW, which means that rehabilitation made an important contribution to patients’ reintegration into the labour market. The effect is therefore not conclusively clarified.

### Limitations and strengths

In interpreting the results, some limitations, strengths, directions for future research and practical implications must be considered. As a first *limitation*, method bias should be taken into account as PIAT contains an observational design. This means that a potential systematic error in the variance of the dependent variable exists owing to the use of only one measurement method. The second limitation pertains to the selection of patients in the T3 follow-up questionnaire and the drop-out of patients due to long questionnaires. It can relatedly be posited that a selection of healthier patients has occurred. Furthermore, based on the here presented results no causal effects can be formulated. Finally, it has to be mentioned that other possible influencing factors, especially the type of treatment, could not be taken into account. We were not able to assess anti-cancer treatments realtime and correlate them with RTW information as well as to validate follow-up patient data with clinical documentation. As we have included information on UICC stage, ASA classification and comorbidities based on clinical documentation in our model, we have at least valid indicators of the stage of disease. In terms of *strengths*, the self-reported dependent variable can be mentioned, as well as the combination of sociodemographic and disease-related variables within one multivariate model which shed light on important issues that should be addressed in future research.

### Implications

Although the study was able to demonstrate significant factors, several *research implications* remain unanswered: i) The possible fluctuating non-linear effect of education on RTW demonstrates the need for longitudinal (panel) studies and differentiated sociodemographic variables in surveys in health services research. In particular, attention to individual or household income and formal education of the partner is critical. This would also control the models for possible non-linear effects and different means of operationalising socioeconomic status [35, 42, 43]. The important role of support from employers and colleagues should also be taken into consideration in future. ii) The empirical interactions of disease-related (UICC stage) and sociodemographic factors (age and having children) [38, 44] have become clearer, as some factors inhibit young women’s RTW. Further qualitative research is needed to investigate the normative values of work and private life [45]. Consequently, patients’ needs appear to be more complex and personal, which should be taken into account in future oncological health services research. iii) In order to concretise this relevant aspect, qualitative research regarding coping strategies and life-sense patterns is necessary. Research on RTW often contains a normative tendency that work represents an important coping resource. The results concerning different age groups presented here indicate that the interaction of sociodemographic and disease-related factors may lead to a decision against RTW. Previous research has shown that the end of employment is also an autonomous decision that is appropriate to the stage of illness and family circumstances [40]. Triangulation of different methods and data sources should be considered in future research proposals. iv) Further possible confounding factors that could not be controlled for in this study might include lifestyle, patient self-management, patients’ health behavior and the above mentioned type of treatment. The theoretical concepts mentioned above appear to be promising, as the findings of previous research have shown that physical activity and sustainable lifestyle or self-management modifications have a beneficial effect on breast cancer prognoses [18, 46, 47]. Additionally, the type of treatment has to be taken into account as different studies show an association with RTW [12, 48, 49]. v) The effect of rehabilitation has not been fully clarified because the multivariate and descriptive results indicate divergent directions of association. Therefore, statistical models that contain organisational and individual variables (multilevel regression model) are needed. A follow-up questionnaire and the use of data from the German National Pension Insurance should prove valuable in future research in order to deepen our understanding of the association between rehabilitation and RTW.

As *practical implications*, two aspects appear to be important. vi) Greater focus should be placed on RTW-supporting healthcare professionals and institutions. It has been highlighted that the “unmet supporting needs” of breast cancer patients are equivalent to unmet informational needs: intervention studies have indicated that unmet information needs can be reduced by small changes, such as physician-patient communication and compliance with the guidelines of treatment, thus helping to minimise health inequalities in healthcare [50, 51]. This should be transferred to unmet supporting needs in order to reduce health inequalities concerning RTW and possibly strengthen e.g. patient self-management. Deficits concerning information and support should increasingly be addressed by social workers, breast care nurses and self-help groups. In particular, informational support from these healthcare professionals would appear to be important, as well as implementation of the RTW topic in organisational structures in breast cancer centres and rehabilitation centres [52]. For the ambulatory setting, this might mean the implementation of an advisory person for RTW questions. Moreover, with implementation of a guiding function between the stationary and ambulatory setting, RTW might be more effectively addressed. vii) Generally, rehabilitation and RTW should be given greater consideration in social policy. Cooperation between health policy and social policy institutions represents a prerequisite for good healthcare.

## Conclusions

This study has highlighted a significant research gap in Germany concerning the determinants of return to work among breast cancer patients. The findings show that significant differences in RTW exist between patient groups, and hence demonstrate the necessity of more effectively addressing RTW issues before, during and after treatment. Longitudinal studies with a follow-up of several years are needed in future research on RTW in Germany. Information and support deficits should be addressed by social services or breast care nurses.

## Additional file

Additional file 1: **Table S4.** Survey items of the descriptive analyses. (DOCX 26 kb)

## Footnotes

1 PIAT: Strengthening patient competence: Breast cancer patients’ information and training needs.
