# Supplementary material for: Sociodemographic and disease-related determinants of return to work among women with breast cancer: a German longitudinal cohort study
Source: BMC Health Serv Res. 2018 Dec 29;18:1000. doi: 10.1186/s12913-018-3768-4 (PMC6311058; doi:10.1186/s12913-018-3768-4)
Supplement: Supplementary file 1 — Table S4. Survey items of the descriptive analyses. (DOCX 26 kb) [file 12913_2018_3768_MOESM1_ESM.docx]

Supplementary file 1:

Table 4:

Survey items of the descriptive analyses

| **Survey item** | **Response trait** |
| --- | --- |
| Have you continued your previous professional activity unchanged? | No  Yes |
| Which working conditions have changed? | More working hours (no/yes)  Less working hours (no/yes)  More work-related stress (no/yes)  Less work-related stress (no/yes)  Higher payment (no/yes)  Lower payment (no/yes)  Range of tasks (no/yes)  Employer (no/yes) |
| Who has supported you concerning RTW? | No support (no/yes)  Health insurance (no/yes)  Pension insurance (no/yes)  Employer (no/yes)  Rehabilitation institution (no/yes) |
